# T1- and ECV-mapping in clinical routine at 3 T: differences between MOLLI, ShMOLLI and SASHA

**DOI:** 10.1186/s12880-019-0362-0

**Published:** 2019-08-01

**Authors:** Julius F. Heidenreich, Andreas M. Weng, Julian Donhauser, Andreas Greiser, Kelvin Chow, Peter Nordbeck, Thorsten A. Bley, Herbert Köstler

**Affiliations:** 1 Department of Diagnostic and Interventional Radiology, University Hospital Würzburg, Oberdürrbacher Str. 6, 97080 Würzburg, Germany; 2 Comprehensive Heart Failure Center, University Hospital Würzburg, Am Schwarzenberg 15, 97078 Würzburg, Germany; 3 Department of Internal Medicine I, University Hospital Würzburg, Oberdürrbacher Str. 6, 97080 Wurzburg, Germany; 4 Siemens Healthcare, Erlangen, Germany

**Keywords:** T1 mapping, MOLLI, ShMOLLI, SASHA, Extracellular volume, 3 T

## Abstract

**Background:**

T1 mapping sequences such as MOLLI, ShMOLLI and SASHA make use of different technical approaches, bearing strengths and weaknesses. It is well known that obtained T1 relaxation times differ between the sequence techniques as well as between different hardware. Yet, T1 quantification is a promising tool for myocardial tissue characterization, disregarding the absence of established reference values. The purpose of this study was to evaluate the feasibility of native and post-contrast T1 mapping methods as well as ECV maps and its diagnostic benefits in a clinical environment when scanning patients with various cardiac diseases at 3 T.

**Methods:**

Native and post-contrast T1 mapping data acquired on a 3 T full-body scanner using the three pulse sequences 5(3)3 MOLLI, ShMOLLI and SASHA in 19 patients with clinical indication for contrast enhanced MRI were compared. We analyzed global and segmental T1 relaxation times as well as respective extracellular volumes and compared the emerged differences between the used pulse sequences.

**Results:**

T1 times acquired with MOLLI and ShMOLLI exhibited systematic T1 deviation compared to SASHA. Myocardial MOLLI T1 times were 19% lower and ShMOLLI T1 times 25% lower compared to SASHA. Native blood T1 times from MOLLI were 13% lower than SASHA, while post-contrast MOLLI T1-times were only 5% lower. ECV values exhibited comparably biased estimation with MOLLI and ShMOLLI compared to SASHA in good agreement with results reported in literature. Pathology-suspect segments were clearly differentiated from remote myocardium with all three sequences.

**Conclusion:**

Myocardial T1 mapping yields systematically biased pre- and post-contrast T1 times depending on the applied pulse sequence. Additionally calculating ECV attenuates this bias, making MOLLI, ShMOLLI and SASHA better comparable. Therefore, myocardial T1 mapping is a powerful clinical tool for classification of soft tissue abnormalities in spite of the absence of established reference values.

## Background

Quantification of myocardial longitudinal relaxation (T1) times is a promising method in cardiovascular magnetic resonance, bearing the ability to characterize myocardial tissue and to identify abnormalities in the context of both acute and chronic events [1, 2]. Evaluation of T1 mapping before and after contrast administration further allows calculation of quantitative myocardial extracellular volume fraction (ECV) maps. This parameter may be superior for the assessment of diffuse extracellular myocardial pathologies like early disease stages of myocardial fibrosis or amyloidosis compared to the commonly used gold standard late gadolinium enhancement (LGE) [3–5].

To date, a variety of T1 mapping sequences is available which use different technical approaches for determination of T1 relaxation times. The most commonly known sequences are the modified look-locker inversion-recovery protocols (MOLLI) [6] and shortened MOLLI (ShMOLLI) [7], as well as the saturation-recovery single-shot acquisition (SASHA) [8]. Each method exhibits specific strengths and weaknesses, which have been studied extensively at 1.5 Tesla. SASHA is reported to yield T1 data with highest accuracy, but MOLLI and ShMOLLI are still superior in terms of precision and image quality [9, 10]. MOLLI and ShMOLLI T1 times were found to be lower with sensitivity to T2 relaxation, magnetic field inhomogeneities [10, 11], inversion efficiency and magnetization transfer (MT) [12, 13]. Sensitivity to T2 and MT may enhance sensitivity to tissue abnormalities [14] while sensitivity to magnetic field inhomogeneities and inversion efficiency may reduce overall specificity.

So far, few studies directly compared in vivo data of different T1 mapping methods at a field strength of 3 T. Reference T1 times and ECV in healthy volunteers with MOLLI and SASHA were just recently published by Weingärtner et al. [15]*.* Teixeira et al. provided native T1 mapping data at 3 T for MOLLI, ShMOLLI and SASHA, demonstrating poor correlation between cardiovascular risk factors and the measured T1 times [16].

The purpose of this study was to evaluate the feasibility and robustness of native and post-contrast T1 mapping methods as well as ECV maps and its diagnostic benefits in a clinical environment when scanning patients with various cardiac diseases at 3 T.

## Material and methods

### Study population

19 patients with clinical indication for contrast enhanced cardiac MRI were examined. In all patients additional native and post-contrast T1 mapping was performed with the three established mapping methods MOLLI, ShMOLLI and SASHA. The study population was comprised of the following cardiac diseases: Anderson-Fabry disease (AFD; *n* = 13; LVH-negative, n = 13; fibrosis, *n* = 4; reduced EF, *n* = 6; confirmed cardiac involvement, *n* = 8), acute myocardial infarction (*n* = 2, positive late enhancement), cardiac amyloidosis (*n* = 1), Friedreich’s ataxia (n = 1), history of cardiac liposarcoma (n = 1), and myocarditis (n = 1). Blood samples were drawn routinely prior to examination to exclude patients with contraindications for MRI and to assess hematocrit for calculation of ECVs. The study was approved by the local IRB and conforms to the ethical guidelines of the 1975 Declaration of Helsinki. Written informed consent was obtained from all study participants prior to inclusion.

### CMR acquisition

All CMR examinations were performed on a clinical 3 T whole body MRI Scanner (MAGNETOM Skyra; Siemens Healthcare, Erlangen, Germany). Myocardial T1 mapping was performed before and in consecutive order 15–20 min after bolus injection of 0.2 mmol/kg gadobenate dimeglumine (Multihance, Bracco Diagnostic, Princeton, United States; 11 patients) or gadoterate meglumine (Dotarem, Guerbet, Aulnay-sous-Bois, France; 8 patients) using the prototype sequences MOLLI, ShMOLLI and SASHA provided by Siemens Healthcare. Each sequence was performed in end-expiration breath holds with the following parameters: TE, 1.04–1.12 ms; echo spacing, 2.6–2.9 ms; acquisition matrix, 256 × 144 (MOLLI, SASHA) and 192 × 144 (ShMOLLI); field-of-view, 270 × 360 mm^2^; voxel size, 1.4 × 1.4 mm^2^ (MOLLI, SASHA) and 1.8 × 1.8 mm^2^ (ShMOLLI); slice thickness, 8 mm; 75% phase resolution, 7/8ths partial Fourier, and GRAPPA rate 2; flip angle MOLLI/ShMOLLI, 35°; flip angle SASHA, 70°; bandwidth, 1085 Hz/pixel. The following sampling schemes were used: native MOLLI: 5(3)3; post contrast MOLLI: 4(1)3(1)2; native ShMOLLI: 5(1)1(1)1; post contrast ShMOLLI: 5(1)1(1)1; native SASHA: 5(3)3; post contrast SASHA: 4(1)3(1)2. Inline motion correction (MOCO) was applied in each sequence.

### T1 mapping analysis

Pixelwise T1 maps were generated online by Siemens Healthcare directly at the scanner. Further segmentation of the T1 maps was done with cvi42® (Circle Cardiovascular Imaging, Calgary, Canada): Endo- and epicardial contours were drawn manually with conservative approach. A further automatic erosion of 15% was applied to avoid accidental inclusion of blood pool or extracardial tissue. The mid-ventricular slice of each patient was segmented into 6 standard segments, which were compared between the different methods. ECV maps were calculated from T1 maps and lab drawn hematocrit values. Segments affected by artifacts were excluded from further analysis. The obtained MRI data was analyzed by allocated radiologists during daily routine. Pathologic segments were identified by consideration of LGE and clinical findings. Segments with inconspicuous LGE and no further pathological alterations were referred to as remote segments.

### Statistics

If not stated otherwise, variables are depicted as mean ± standard deviation (SD). Comparison of techniques was statistically analyzed by a paired non-parametric Wilcoxon test. Statistical analysis of pathologic and remote segments was performed by an unpaired non-parametric Mann-Whitney-U test. Only two-sided tests were used and *p* values *p* <  0.05 were considered statistically significant (*).

## Results

**Fig. 1:**
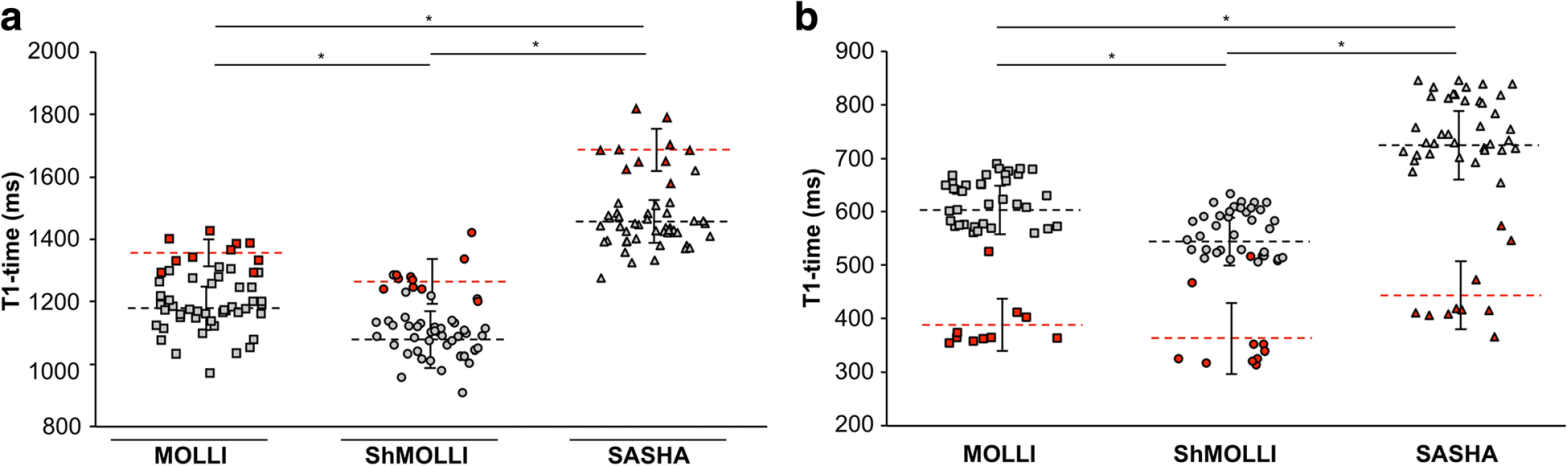
Comparison of absolute myocardial T1 times in a heterogeneous study population. Quantification with MOLLI and ShMOLLI yielded significantly lower native (**a**) and post-contrast T1 times (**b**). T1 times from pathologic segments (red) were clearly distinguishable from remote segments (grey). Data is expressed as absolute T1 times (ms) from all measured segments and mean ± SD. * Significant difference (*p* < 0.05) between pathologic and remote segments

19 patients with cardiac diseases underwent pre- and post-contrast T1 mapping with the three mapping sequences MOLLI, ShMOLLI and SASHA. Pathologic lesions were identified by consideration of T1 relaxation times and LGE and data of the three pulse sequences was compared (Fig. 1, Table 1). Mean T1 relaxation time of remote myocardium was lowest with ShMOLLI (1076 ± 100 ms) compared to MOLLI (1175 ± 72 ms) and SASHA (1460 ± 67 ms), with broadest distribution found in ShMOLLI (outlined by standard deviation SD, Fig. 1a). Native T1 time was remarkably higher in pathologic segments (ShMOLLI: 1264 ± 72 ms; MOLLI: 1356 ± 43 ms; SASHA: 1687 ± 68 ms; solid red). In line with this, post-contrast T1 mapping allowed differentiation of pathologic segments by significantly shorter T1 times (ShMOLLI: 366 ± 74 ms; MOLLI: 388 ± 49 ms; SASHA: 444 ± 63 ms) than in remote myocardium (ShMOLLI: 541 ± 49 ms; MOLLI: 599 ± 53 ms; SASHA: 720 ± 68 ms; Fig. 1b).

**Table 1:** Comparison of myocardial/blood T1 times and ECV measured with MOLLI, ShMOLLI and SASHA at 3 T

	MOLLI	ShMOLLI	SASHA	*p*-value
Native				
T1-time [ms]				
Myo				
remote	1175 ± 72	1076 ± 100	1460 ± 67	*
pathologic	1356 ± 43	1264 ± 72	1687 ± 68	*
Blood	1917 ± 96	1798 ± 77	2214 ± 101	*
Post-contrast				
T1-time [ms]				
Myo				
remote	599 ± 53	541 ± 49	720 ± 68	*
pathologic	388 ± 49	366 ± 74	444 ± 63	*
Blood	404 ± 93	379 ± 86	424 ± 93	*
ECV [%]				
remote	25.7 ± 2.9	27 ± 3.4	22.9 ± 3.2	*
pathologic	43.7 ± 4.9	43.3 ± 4.9	40.3 ± 5.3	*

Data is expressed as mean of all measured segments. Statistical significance was analyzed for MOLLI vs. SASHA and ShMOLLI vs. SASHA. *, *p* <  0.05

**Fig. 2:**
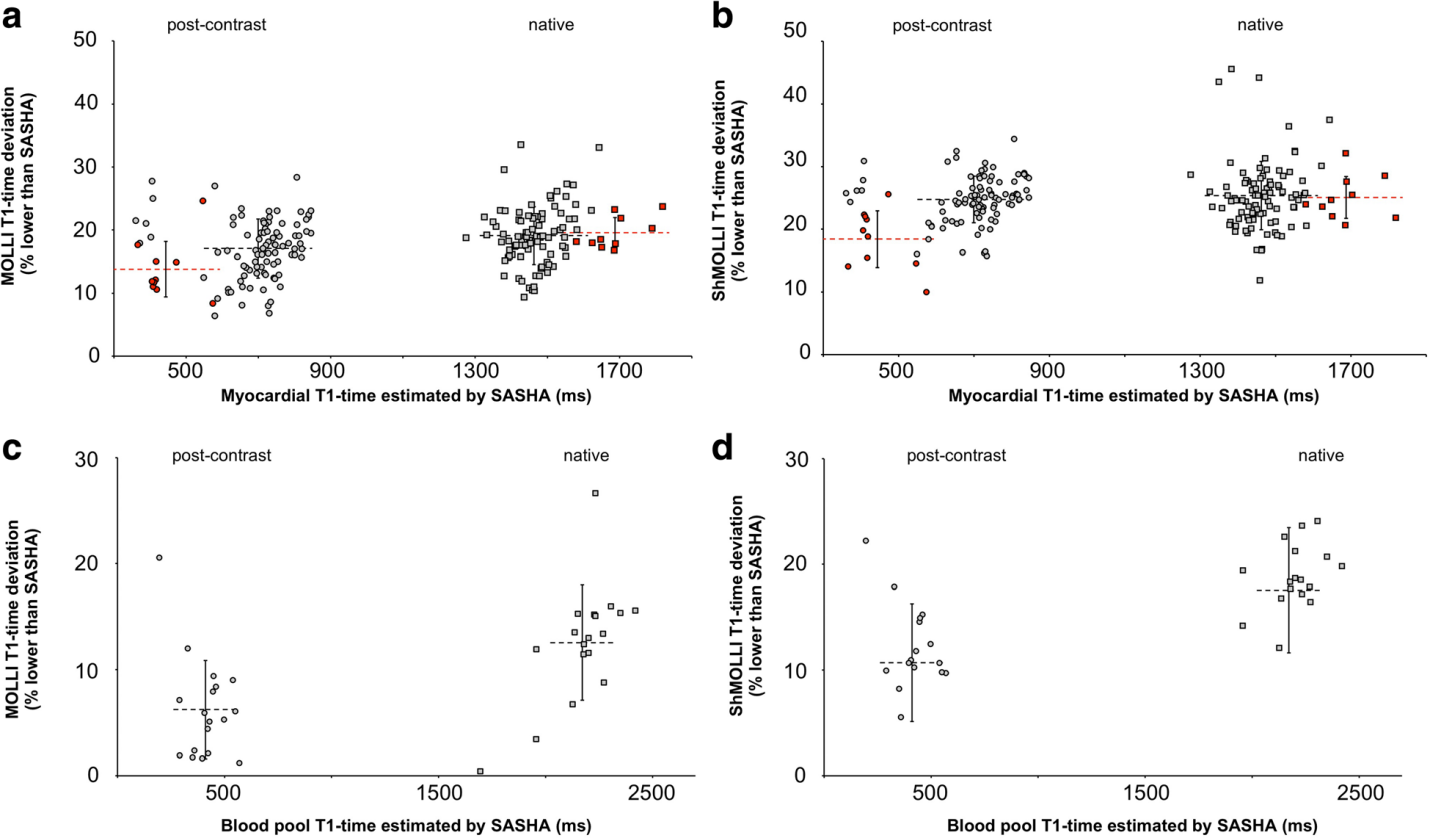
Percental T1 deviation of pre- and post-contrast MOLLI and ShMOLLI in myocardium and blood. T1 error in myocardium is less prominent in MOLLI (**a**) than ShMOLLI (**b**) but no significant difference could be observed between pre- and post-contrast T1 error. T1 error was comparable in pathologic (red) and remote segments (grey). T1 deviation in blood was highly dependent on T1 time and significantly different pre- and post-contrast T1 deviation were obtained with MOLLI (**c**) and ShMOLLI (**d**). Data is expressed as percental deviation of respective T1 time from SASHA in %. Mean ± SD are indicated

The presented in vivo study revealed systematically shorter native and post-contrast T1 times by MOLLI and ShMOLLI compared to SASHA (Fig. 2). Regardless of being performed under native or post-contrast conditions, deviation of MOLLI T1 times in remote myocardium was similar (19.5 ± 4.4% vs 16.8 ± 4.6% lower, respectively; Fig. 2a and Table 2). When performed with ShMOLLI, the observed T1 difference was more prominent (25.3 ± 5.4% and 24.8 ± 3.7% lower; Fig. 2b). With both inversion-recovery methods native T1-times were similarly different in pathologic (red) and remote myocardium compared to SASHA. Deviation of T1 values was less pronounced in pathologic myocardium at post-contrast conditions (MOLLI: 13.8 ± 4.4%; ShMOLLI: 18.4 ± 4.5% lower; Fig. 2a, b).

**Table 2:** Systematic T1 error in inversion-recovery methods MOLLI/ShMOLLI compared to SASHA

	native	post-contrast	*p*-value
Myocardial T1 error (% lower than SASHA)			
MOLLI	19.5 ± 4.4	16.8 ± 4.6	< 0.05
ShMOLLI	26.3 ± 6.3	24.8 ± 3.7	< 0.05
Bloodpool T1 error (% lower than SASHA)			
MOLLI	13.3 ± 4.8	5.4 ± 6.1	< 0.05
ShMOLLI	18.7 ± 3.1	11.5 ± 5.1	< 0.05

Assessed errors in myocardial tissue are comparable between native and post-contrast mapping from MOLLI and ShMOLLI, independently from respective T1 times. Blood T1 error exhibits significant dependence on respectively measured T1 time. Data is expressed mean percental deviation ± SD from T1 time measured with SASHA. *p* < 0.05 is considered statistically significant

T1 differences using MOLLI or ShMOLLI compared to SASHA were less prominent when measuring T1 of the blood pool (Fig. 2c, d). Deviation of T1 times was higher under native conditions (MOLLI: 12.5 ± 7.2%; ShMOLLI: 17.5 ± 6%) than after administration of contrast agent, where T1 difference was less prominent (MOLLI: 6.2 ± 4.6%; ShMOLLI: 10.6 ± 5.5%; Fig. 2c, d and Table 2).

**Fig. 3:**
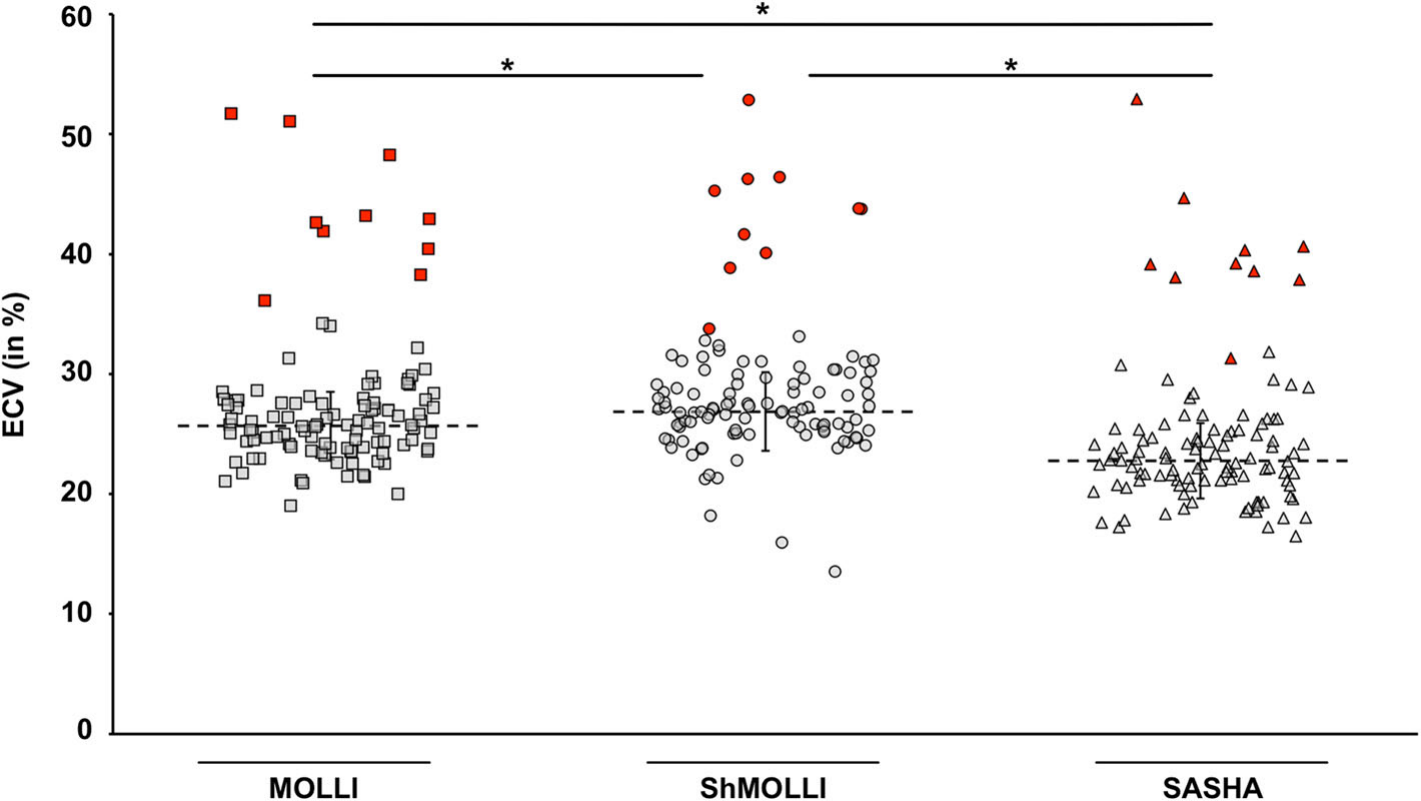
Comparison of determined ECV with MOLLI, ShMOLLI and SASHA. MOLLI and ShMOLLI give significantly higher ECV values compared to SASHA. Pathologic segments (red) can clearly be distinguished from remote segments (grey). Data is expressed as volume in % and mean ± SD are indicated. *, p < 0.05

**Fig. 4:**
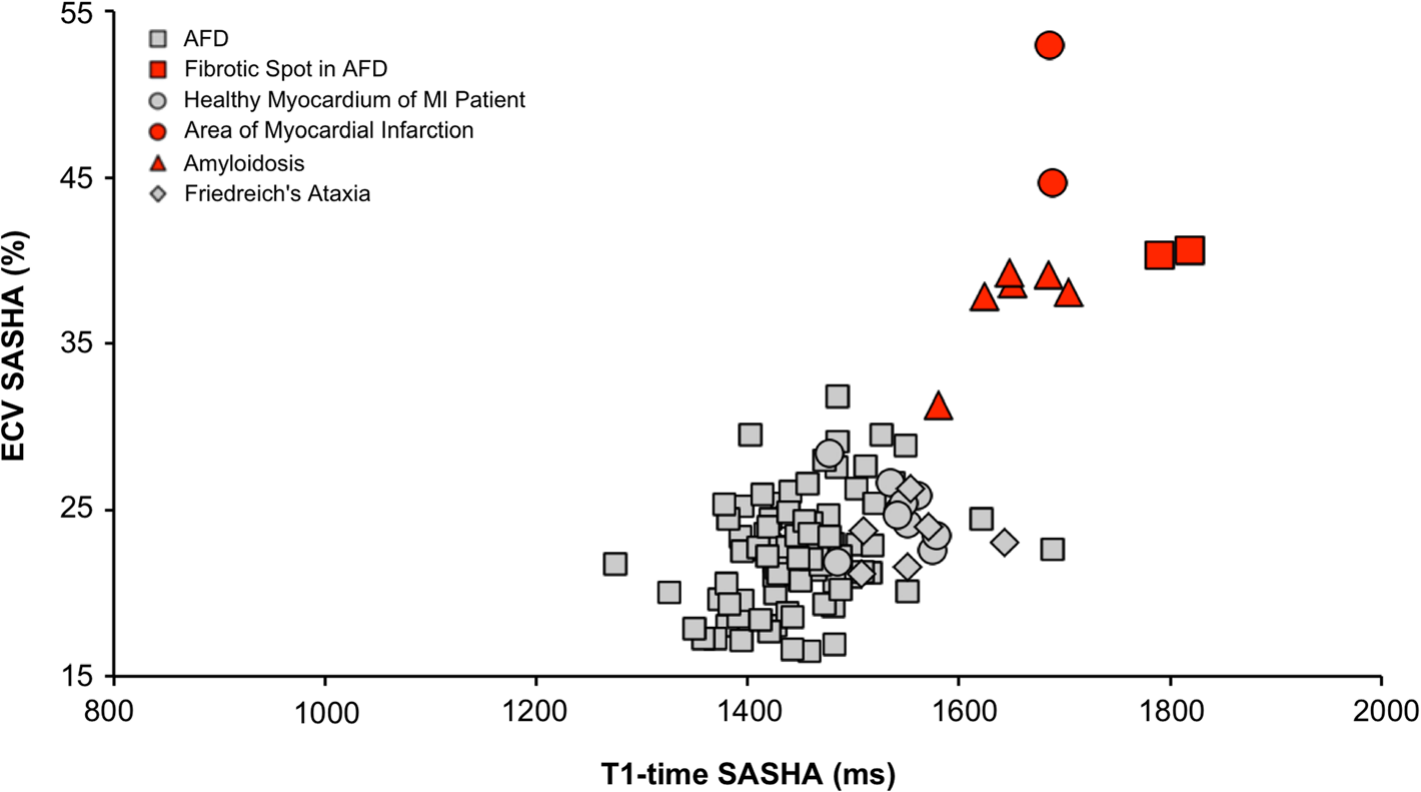
Consideration of native and post-contrast T1 data allows demarcation of remote and pathologic myocardium. Native and post-contrast T1 times were acquired with SASHA and ECV was calculated. Several pathological and remote segments show overlapping T1 times but can be subsequently distinguished when additionally also considering ECV data

The ECVs from MOLLI, ShMOLLI and SASHA, calculated as described before [3, 17], were compared with values from pathologic and remote segments (Fig. 3). Mean ECV from remote myocardium was estimated higher with ShMOLLI (27 ± 3.4%) and MOLLI (25.7 ± 2.9%) compared to SASHA (22.9 ± 3.2%), meaning an overestimation of 12.8% by MOLLI and 17.9% by ShMOLLI. ECV of pathologic lesions significantly exceeded ECVs in remote myocardium (MOLLI: 43.7 ± 4.9%; ShMOLLI: 43.3 ± 4.9%; SASHA: 40.3 ± 5.3%). Interestingly, ECVs from pathologic myocardium were only 8.4% higher with MOLLI and 7.6% higher with ShMOLLI compared to SASHA (Table 2). Combined consideration of both, ECV and native T1 times, allows even better differentiation of pathologic and remote myocardium (Fig. 4).

**Fig. 5:**
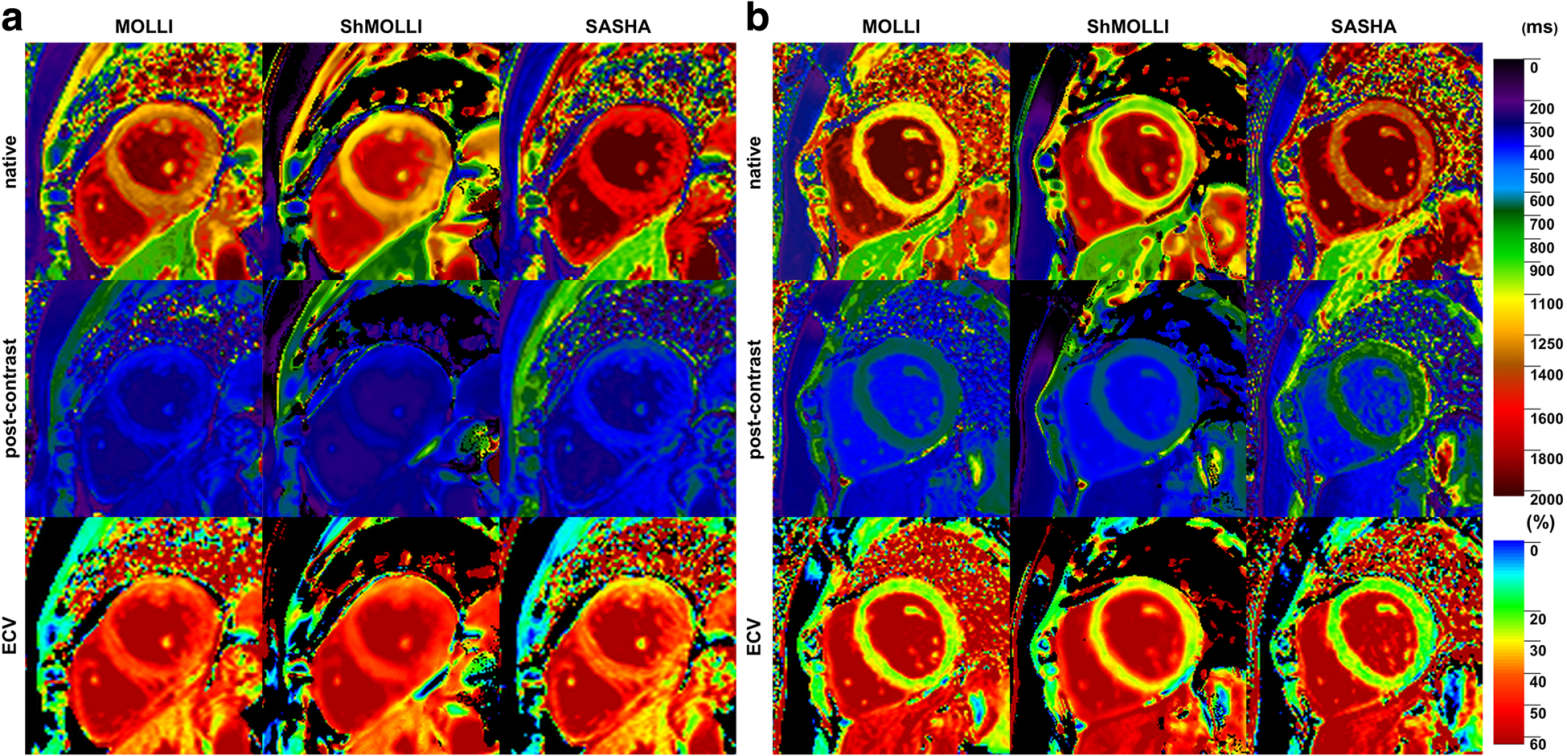
T1 and ECV color maps give clear hint for presence of cardiac pathology. T1 and ECV maps from a patient suffering from Amyloidosis (**a**) and Anderson-Fabry disease (**b**) were generated with cvi42®. Prolonged native-, shortened post-contrast T1 times and elevated ECV are clearly recognizable in (**a**) as sign of extracellular amyloid deposition. In comparison, ECV of a patient with intracellular Anderson-Fabry disease remains unaffected (**b**)

Figure 5 compares T1 maps and ECV from a patient with myocardial amyloidosis (a) and with Anderson-Fabry disease (b). Significantly elevated ECV was found in amyloidosis by all of the three techniques (MOLLI: 41 ± 2%; ShMOLLI: 43 ± 2%; SASHA: 37 ± 2%). ECV elevation in comparison to a patient with Anderson-Fabry Disease is shown in (b). Native T1 maps from patient (a) exhibit significantly prolonged T1 times compared to patient (b).

## Discussion

In this study we analyzed pre- and post-contrast data from patients with myocardial diseases and an indication for contrast-enhanced magnetic resonance imaging. Cardiac T1 mapping was performed with three recognized mapping techniques MOLLI, ShMOLLI and SASHA and T1 relaxation times of the different pulse sequences were compared with each other. We aimed to analyze the obtained differences in T1 relaxation times and ECV maps using the three T1 mapping methods and to further investigate the feasibility and robustness of cardiac T1 mapping during clinical routine examination.

Pre- and post-contrast T1 mapping with different mapping techniques at 1.5 T was addressed before [18]. At 3 T native T1 mapping with MOLLI, ShMOLLI and SASHA was analyzed by Roujol et al. as well as Teixeira et al. [9, 16], while systematic analyzes of pre- and post-contrast MOLLI, ShMOLLI and SASHA is still sparse.

To be noted, native T1 times from remote myocardium found in our study (MOLLI: 1175 ± 72 ms; ShMOLLI: 1076 ± 100 ms; SASHA: 1460 ± 67 ms) are slightly shorter compared to previously published data [15, 16, 19–21]. This might be explained by the composition of the presented study population. Which was made up predominantly by patients with mild form of genetically confirmed Anderson-Fabry disease. Sado et al. and Thompson et al. showed that intracellular myocardial sphingolipid deposition as part of Anderson-Fabry disease leads to significantly shortened native T1-times [22, 23]. Pica et al. showed that T1 mapping in Fabry disease is a reproducible technique with shorter myocardial T1 times at 1.5 T [24].

T1 mapping subsequent to contrast bolus injection provides post-contrast T1 times and thereby gives further information for tissue characterization. Recently, Weingärtner et al. published reference values for pre- and post-contrast T1 times using the mapping sequences MOLLI and SASHA [15]. Post-contrast T1 times from our study fit well with these data, but more importantly, similar systematic differences between the mapping sequences were observed pre- and post-contrast application.

SASHA may provide the most accurate measurement of T1 relaxation time due to its robustness to confounders [10]. Therefore, we assessed SASHA as reference sequence in this study, and compared the ratio of T1 times from MOLLI and ShMOLLI to SASHA. Previous phantom studies at 1.5 T, 3 T [9, 10, 16] and in vivo T1 mapping by others revealed relevant systematic T1 errors in MOLLI and ShMOLLI [12, 13]. Furthermore, the inversion recovery sequences exhibited a sensitivity to T2 relaxation, inversion efficiency and magnetization transfer [12, 13]. We demonstrated similar systematic T1 underestimation in vivo by MOLLI and ShMOLLI in our study population. Greater T1 errors for MOLLI and ShMOLLI were found in the myocardium compared to the blood pool, consistent with greater T1 underestimation in the short T2 myocardium (~ 45 ms) than the longer T2 blood pool (~ 275 ms, [25]). Messroghli et al. reported increased MOLLI T1 error with longer T1 times [6, 7, 26]*.* In line with this, our data demonstrates that T1 deviation of the blood pool was 2.5 fold higher under native conditions (long T1) compared to post-contrast conditions (short T1; Table 2).

Post-contrast T1 is strongly influenced by contrast agent dosage and timing. Myocardial and blood T1 times increase with the elapsed time after contrast bolus application, whereas the myocardial partition coefficient λ, a ratio of ΔR_myo_ and ΔR_blood_ remains constant [17]. When analyzing λ, confounders as contrast agent and elapsed time after contrast injection can be excluded. However, T2 relaxation bias of T1 still affects estimation of λ. In combination with laboratory-tested hematocrit, λ is regularly used for determination of extracellular volume fraction (ECV) [3, 17]. ECV is a powerful parameter with important prognostic value, which facilitates detection of diffuse myocardial pathologies such as early fibrosis or amyloidosis. Our data provided ECV values which are in good agreement with data from literature for MOLLI [15, 27, 28] and SASHA at 3 T [15]. As mild Anderson-Fabry disease is an intracellular pathology where ECV remains unaffected, obtained ECV values from mild disease can be considered as quite comparable to those from healthy patients [29].

It has been addressed before that MOLLI and ShMOLLI mapping sequences provide T1 values that systematically overestimate ECV compared to SASHA [15]. This is not surprising since ECV estimated by T1 mapping is a calculated ratio from myocardial λ and hematocrit [3, 17], which subsequently is biased by the described T2-dependent T1 estimation errors. Thereby, calculation of ECV with erroneous T1 times from myocardium and blood generally results in overestimation of ECV by inversion recovery sequences. Figures 3 and 4 demonstrate the importance of contrast-enhanced T1 mapping, since pathologic segments are clearly distinguishable from healthy myocardium when considering both, native T1 times and ECV.

Interestingly, Robson et al. supposed that confounds of magnetization transfer and T2, which bias the T1 measurement in MOLLI, may enhance the sensitivity to disease detection, as the T1 bias further increases because of tissue abnormalities [14]. In this case, inversion recovery techniques as MOLLI and ShMOLLI would be to prefer over SASHA, as discrimination between healthy and pathologic myocardium would be enhanced. Interestingly, our data shows opposite results: T1 values of pathologic myocardium indeed showed increased bias as compared with SASHA T1 values, but exhibited fewer differences as compared with healthy myocardium, leading to a closer overlap between healthy and pathologic and remote myocardium (Figs. 1 and 2a, b). This stands in contrast to Robson et al.*,* as we did not observe enhanced changes of native T1 times in pathologic segments by MOLLI and ShMOLLI, but rather an increased T1 difference with SASHA (Fig. 1a). Considering the presented data T1 mapping with SASHA might be preferable, since the proposed advantage of T1 bias in inversion recovery sequences is questionable.

MOLLI and ShMOLLI systematically underestimated T1 times in our patient population compared to SASHA. The observed T1 error was less prominent in the blood compared to the myocardium. Furthermore, T1 error in blood exhibited a dependence on length of actually measured T1 time, with increasing deviation upon increasing T1 times. Biased T1 times led to higher estimation of ECVs with MOLLI and ShMOLLI compared to SASHA. Interestingly, post-contrast error between MOLLI/ShMOLLI and SASHA was less prominent in remote than in pathologic myocardium and in line with this, pathologic myocardium could be distinguished from remote tissue independently from the used sequence. Focal as well as diffuse myocardial pathologies could be identified, though absolute T1 times were not comparable among sequences. Our results indicate that T1 mapping is an efficient tool for evaluation and comparison of tissue characteristics with promising feasibility and robustness in clinical application using either technique. However, a gold standard needs to be established, as mapping data from different sequences is not readily comparable.

This study has several limitations. The patient population is mixed, but heavily dominated by patients with Anderson-Fabry disease, a relatively rare disease which does not allow a generalization on typical clinical population. Furthermore, in 5 patients AFD was genetically confirmed, but no proof for cardiac involvement was found in MR imaging, which assigns these patients an early stage of disease. T1 times of these patients were minimally, but not significantly longer as compared with T1 of remote myocardium in patients with AFD and cardiac involvement. More importantly, the above presented differences between MOLLI, ShMOLLI and SASHA were observed independently from cardiac involvement in AFD patients. Remote (healthy) and pathologic myocardium was determined by consideration of LGE, however, LGE-free segments cannot necessarily be considered as healthy myocardium as shown before [22, 23]. Further, this study lacks a healthy control group since we did not recruit healthy volunteers for contrast enhanced MRI, yet, for the intraindividual differences of the three mapping techniques a healthy control group is dispensable. Furthermore, image acquisition was subject to time constraints because of clinical routine.

CMR-protocols were modified during the time of data acquisition, which is why T1 mapping was performed either with gadobenate dimeglumine (Multihance) or gadoterate meglumine (Dotarem). To overcome these inconsistencies, we did not compare absolute T1 times, but calculated the ratios between MOLLI/ShMOLLI and SASHA. These limitations may be also considered as a strength since robustness and error proneness of T1 mapping sequences were evaluated. Furthermore, T1 mapping was performed after a single bolus contrast-agent application with MOLLI, ShMOLLI and SASHA in consecutive order, which in turn may lead to higher post-contrast T1 times and lower ECV with SASHA.

It is important to single out, that the focus of this study was not to establish reference values for T1 times at 3 T or to proof the deviance of T1 relaxation times between remote and pathologic myocardium. The aim was rather to analyze differences of the three T1 mapping sequences amongst each other before and after contrast bolus administration, the obtained ECV maps and moreover the robustness during clinical routine.

## Conclusion

T1 mapping at 3 T is a feasible and robust method for characterization of myocardial tissue during clinical routine. Systematic deviation of T1 relaxation times from MOLLI, ShMOLLI compared to SASHA occurred in clinical routine. Post-contrast mapping allows the generation of ECV maps, which are similarly biased by deviation of T1 estimation. Combined analysis of ECV and native T1 times might enhance the diagnosis of myocardial pathologies. SASHA may provide the most accurate absolute T1 times, but reference values are needed for each mapping technique in order to bring T1 mapping into routine clinical CMR imaging.

## Data Availability

The datasets generated and/or analysed during the current study are not publicly available as MRI data and DICOM headers contain patient information. Data can be obtained on reasonable request from the corresponding author.
